# Exploring associations between oral health measures and oral health-impacted daily performances in 12–14-year-old schoolchildren

**DOI:** 10.1186/s12903-022-02341-9

**Published:** 2022-07-23

**Authors:** Mir Faeq Ali Quadri, Mohammad Abdullah Alwadani, Khalid Mohammad Talbi, Reem Ali Ahmed Hazzazi, Rami Hassan Abdullah Eshaq, Faisal Hussain Jaber Alabdali, Mohammed Hassan Mofareh Wadani, Gianluca Tartaglia, Basaruddin Ahmad

**Affiliations:** 1 Dental Public Health, Department of Preventive Dental Sciences, College of Dentistry, Jazan University, Jizan, 45142 Saudi Arabia; 2 Department of Pediatric Dentistry, Jazan Specialized Dental Centre, Ministry of Health, Jazan, Saudi Arabia; 3 Ministry of Health, Riyadh, Saudi Arabia; 4 College of Dentistry, Jazan University, Jizan, 45142 Saudi Arabia; 5 Department of Biomedical, Surgical and Dental Sciences, School of Dentistry, University of Milan, 20100 Milan, Italy; 6 UOC Maxillo-Facial Surgery and Dentistry Fondazione IRCCS Cà Granda, Ospedale Maggiore Policlinico, Milan, Italy; 7 School of Dental Sciences, Health Campus, Universiti Sains Malaysia, 16150 Kubang Kerian, Kelantan Malaysia

**Keywords:** Oral health-related Quality of Life, Schoolchildren, Oral diseases, Oral health measures, Association

## Abstract

**Background:**

Oral health-related quality of life, a prominent topic in dentistry, has been studied extensively. However, the comparison between various self-perceived and clinical oral health measures still needs to be explored. The purpose of the current study is; first, to report the self-perceived and clinically examined oral health measures that are associated with the oral impacts on daily performances. Second, to identify the oral health measure that best predicts greater oral impact scores. Third, to investigate the difference in findings related to the disease experience measures and the treatment measures.

**Methods:**

A cross-sectional study was carried out on children aged 12–14 years. The prevalence, frequency, and oral impact scores of each daily performance were calculated. Thirteen self-perceived OH conditions were assessed. Clinically examined oral health measures included gingival health, oral hygiene status, DMFT, DT, MT, and FT scores and, one or more decay (1 + D), missing (1 + M) and filled (1 + F) teeth. Simple linear and multiple linear regressions were carried out to report the associations.

**Results:**

At least one oral health impacted daily performance was reported by 40% of the total sample of schoolchildren (N = 700). Based on the magnitude and precision of adjusted regression coefficients (RC), decay severity (DT) was identified as a better predictor of a greater oral impact score with regression coefficient values ranging between 0.3 (social contact) and 2.4 (1 + performance). Contrariwise, MT and FT components of DMFT were associated with lower oral impact scores. The self-perceived measures were also associated with oral impact scores and presented similar findings to that of the clinical oral health measures.

**Conclusions:**

Untreated decay significantly impacted daily performances, especially eating, sleeping, studying, and social contact. The findings are of importance to public health practitioners for reporting, treating, and preventing oral health problems in children, and eventually contributing to better oral health-related quality of life.

## Background

Oral health-related quality of life (OHRQoL), involving participant-based outcome (PBO) measures, is a prominent topic in dentistry [1]. This assessment compliments the biomedical concept of oral diseases by considering a wider biopsychosocial model, thereby presenting the overall health and well-being of individuals. In adolescent-aged schoolchildren, the Child Perception Questionnaire (CPQ), the Child Oral Health Impact Profile (COHIP), and the Child Oral Impacts on Daily Performances (C-OIDP) are commonly used OHRQoL assessment tools. These tools avoid proxy measures i.e. collect information straight from children instead of parents/caregivers and demonstrate high methodological quality (EMPRO: evaluating the methodological quality of each study focused on measurement properties) [2]. Between them, the C-OIDP assessment tool is shorter with less items to respond, constituting a lower participant burden, and is, therefore, easier in collecting information on the prevalence, frequency, and severity of oral impacts while interviewing a larger study sample [3].

The C-OIDP is based on the concept that oral health (OH) conditions negatively impact the specific daily performances of children, such as; eating, speaking, cleaning teeth, relaxing including sleeping, smiling, laughing and showing teeth without embarrassment, maintain emotional state, study including going to school and doing homework, and contact with other people [4]. Eighteen studies exhibited the test–retest reliability with intraclass correlation values ranging from 0.70 to 0.98, and five studies confirmed the internal consistency with Cronbach’s alpha values ranging from 0.79 to 0.91 [2, 5]. Moreover, this tool has been used in reporting the OHRQoL of children from diverse ethnic and language backgrounds [4, 6–8].

Despite the extensive application, impact of OH measures on specific daily performances and the overall C-OIDP score still needs to be explored. The earlier studies that examined the impact of active caries did not investigate the extent of its impact [9, 10]. Further, it is unclear from the existing reports, which of the clinical and/or self-perceived OH measures is a better predictor of the impacted daily performances. Besides, there is no expansive information on the OHRQoL of children in Saudi Arabia using the C-OIDP tool, as the first study only involved the boys sample [11]. Next, none of the earlier reports had differentiated the findings related to disease experience measures and treatment measures.

Thus, if the result of an association between oral impact score and clinical caries measures, such as having at least one caries tooth (≥ 1), is comparable to self-perceived tooth decay, and if the caries experience measure using DMFT has a similar impact to its components, the DT, MT and FT, then the findings of OHRQoL outcomes may contribute better in policy-making and -application [12]. To address these gaps in understanding, the objectives were; first, to report the self-perceived and clinically examined oral health (OH) measures that are associated with the oral impacts on daily performances. Second, to identify the OH measure that best predicts greater oral impact scores based on the magnitude of effect size and precision. Third, to investigate the difference in findings related to the disease experience measures and the treatment measures.

## Methods

### Study design and sampling issues

A cross-sectional study was carried out in children aged 12–14 years attending the government-funded schools in Jazan, Saudi Arabia. The sample size calculation, based on the proportion of untreated caries [9, 13] showed that 638 subjects were needed at 5% precision and 95% confidence level; and was rounded up to N = 700 to account for 10% loss due to refusals, dropouts and missing data. Because of the gender-segregated schooling system (number of schools: boys = 15; girls = 10) and to minimize the cost and number of visits to the schools, a stratified and randomized cluster sampling method was adopted. The total sample was first stratified by gender (n_girls_ = n_boys_ = 350) and all children, with no medical conditions, aged 12–14 years in the randomly selected schools were included. The number of schools required was determined by dividing the stratified sample by the average number of targeted children (n_12–14 years_ = 90 in a school); thus 4 schools for each gender (350 ÷ 90 = 3.89 ≈ 4).

### Ethical issues and calibration process

Data collection was carried out after the ethical approval from the Jazan University (letter dated: COD-JU, 7 March 2017) and, permissions from the Ministry of Education (letter dated: 26-03-2017) and selected schools were obtained. Informed consent forms were passed to the parents through the class teachers. The signed forms were collected and verified by the children on the examination day. The investigator (MFAQ) and three male students attended the boys’ school and, three female students attended the girls’ school because male visitors are not permitted. The students were trained and calibrated before the commencement of the study to carry out oral clinical examinations and questionnaire administrations. MFAQ carried out the clinical examinations in the male schools and the female final year students were supervised by a graduate intern while they carried out the clinical examinations in the female schools. The clinical calibration involved examination of caries, gingival and plaque statuses of six index teeth in 30 children, and the inter-examiner reliability including the investigator was good (unweighted Kappa = 0.84 ± 0.03) [14, 15].

### Data collection process

The data collection process was carried out in a succession of the schools using the same protocol and assisted by six native Arabic speaking final year dental students who were affiliated with the university. A batch of ten children were brought to the examination room allocated by the school authority from their classrooms. After the consent was verified, they were asked to complete a set of self-administered questionnaires under the supervision of the final year dental students. Then, an oral examination was carried out using disposable probes and mouth mirrors with the child seated on a normal chair under a light-emitting diode (LED). Children who needed dental treatment were advised and provided with a reference letter to visit the dental clinics at the university. The children were returned to their class before a new batch was brought in, and a similar procedure was followed in all the selected schools.

### Data collection tools and study variables

The administered questionnaires included information on demographic charateristics (age, location, parental education), teeth cleaning tools (miswak, toothbrush, both), frequency of cleaning (irregular, once/day, ≥ twice/day) and dental visits in the past year (never, once, ≥ 2 twice). A self-perceived OH assessment tool adapted from a previous report was included, and information on the presence or absence of a range of OH conditions that occurred in the past three months was collected [4]. Then, the children completed a validated Arabic version of the C-OIDP inventory, which measured the oral impact on eight daily performances in the past three months relating to eating, speaking, cleaning teeth, relaxing/sleeping, emotion, smiling, study, and social contact [4, 16]. It asked whether any of the daily performances were impacted, and if yes, indicated the frequency (not at all = 0, once = 1, twice = 2, thrice or more = 3) and severity (not at all = 0, little = 1, moderate = 2, severe = 3) of occurrence. The prevalence and score-based measures for the individual and overall daily performances were derived from the instrument. The *prevalence* of impact for each daily performance was derived from the frequency of occurrence ≥ 1. The overall prevalence (1 + performance) was defined as having at least one daily performance impacted with a frequency of at least ≥ 1. The *oral impact score* of each daily performance was calculated by multiplying the scores for frequency and severity, which ranges from 0 to 9 [4, 17]. However, the range of impact depended on the response from the study participants, and if one of the options on the Likert scale (either for frequency or for severity) was valued at 0, then the range was considered from 0 to 6 (impacted only) [4]. The *overall impact score* was calculated by dividing the total *oral impact score* of the eight performances by 72 and then multiplying it by 100. In both impact scores, a greater value indicates a greater impact on the performance. The clinical oral examination was carried out based on the WHO recommended methods [18]. The comprehensive score of Decayed Missing and Filled Teeth (DMFT), the specific score of decayed (DT) missing (MT) and filled (FT) teeth relating to the severity of the condition and, one or more decayed (1 + D), missing (1 + M) and filled (1 + F) tooth relating to the presence or absence of the condition were recorded. The gingival health and oral hygiene status were assessed using the Gingival Index (normal, mild inflammation and moderate/severe inflammation [19] and Plaque Index (absence of plaque, mild accumulation, and moderate/severe accumulation) [20], respectively.

### Statistical analysis

The descriptive analyses of all variables were carried out separately by gender. Chi-square test and t test were used for comparison between the gender. Cochrane-Armitage test for trend was used for the frequency of brushing, dental visit, and plaque and gingival indices to examine the effect of increasing exposure. Data from both genders were then combined in the analysis for C-OIDP. The proportion of children reported with the prevalence, frequency, and severity of impacts was computed, followed by the oral impact scores (OIS). Simple linear regressions examined the association of the OH measures (clinical and self-perceived) with the oral impact scores of 1 + performance and other daily performances. For significant OH measures, multiple linear regression analyses were carried out to determine the adjusted effect size by adding the covariates (oral health behaviours and demographic characteristics) using the backward selection method at p_inclusion_ = 0.05, and p_removal_ ≥ 0.1. The relative effect size (regression coefficient; RC) and precision of the confidence intervals from the multiple linear regressions were contrasted to determine the OH measure that was consistent and best predicted the impact on the daily performances. All analyses were carried out in IBM SPSS v24 at a 5% significant level.

## Results

A total of 720 children were invited to participate in the study. However, 9 boys and 11 girls did not provide the signed consent, therefore the final sample size was 700 (N) consisting of 51.6% boys and 48.4% girls. The mean age was 12.8 years (SD = 0.79), and the proportion of children from the 12-year-old age group was greater (44.6%), followed by 13-year-old (32.9%) and 14-year-old (22.6%). More girls had higher educated mothers and less educated fathers (< 0.05). Most children used the toothbrush to clean their teeth but; more boys used miswak exclusively, cleaned their teeth, and visited the dentist less frequently than the girls (Table 1).

**Table 1:** Frequency distribution of socio-demographic characteristics and oral hygiene behaviors, by gender (N = 700)

Variable	Frequency N = 700	Gender		p value
		Boys 361 (51.6%)	Girls 339 (48.4%)	
*Age*				0.02
12 years	312 (44.6%)	155 (42.9%)	157 (42.5%)	
13 years	230 (32.9%)	135 (37.4%)	95 (28.0%)	
14 years	158 (22.6%)	71 (19.6%)	87 (26.6%)	
Mean (SD)	12.78 (0.79)	12.77 (0.76)	12.79 (0.82)	0.7
*Location*				0.1
Urban	294 (42.0%)	140 (38.7%)	154 (45.4%)	
Rural	406 (58.0%)	221 (61.2%)	185 (54.6%)	
*Mother’s education*^1^				0.01
Uneducated	40 (5.7%)	30 (8%)	10 (2.9%)	
Secondary School	151 (21.6%)	82 (22.7%)	69 (20.3%)	
High school	362 (51.7%)	180 (49.8%)	182 (53.7%)	
Graduate	147 (20.7%)	69 (19.4%)	78 (23%)	
*Father’s education*^2^				0.01
High school	331 (47.3%)	153 (42.4%)	178 (52.5%)	
Graduate	320 (45.7%)	185 (51.2%)	135 (39.8%)	
Post graduate	49 (7.0%)	23 (6.4%)	26 (7.6%)	
*Oral hygiene tools*				
Miswak only	99 (14.1%)	66 (18.3%)	33 (9.7%)	< 0.001^3^
toothbrush only	566 (80.9%)	272 (75.3%)	294 (86.7%)	
Both	35 (5.0%)	23 (6.4%)	12 (3.5%)	
*Frequency of teeth cleaning*				
Irregular	150 (21.4%)	60 (16.6%)	90 (26.5%)	< 0.001^3^
Once/day	375 (53.6%)	212 (58.7%)	163 (48.0%)	
≥ Twice per day	175 (25.0%)	89 (24.6%)	86 (25.4%)	
*Last visit to a dentist (in the past year)*				
Never	24 (3.4%)	0	24 (7.1%)	< 0.001^3^
Once	294 (42%)	176 (48.7%)	118 (34.8%)	
Twice or more	382 (54.6%)	185 (51.2%)	197 (58.1%)	

^1^Highest level of education in mothers was graduate

^2^Lowest level of education in fathers was secondary school

^3^Cochran-Armitage tests for trend. For this the %—row, alternatively—just chi^2^

The prevalence of caries experience (DMFT ≥ 1) in the sample was 34.4% with a mean DMFT score of 3.8 (SD = 0.75). The DMFT and FT scores were significantly lower in boys than girls but by a very small difference. There were more girls with missing and filled teeth and, more severe plaque and gingival statuses compared to boys (Table 2). In the self-perceived OH conditions, more girls than boys reported having tooth decay, tooth extraction, and halitosis and; more boys than girls had discoloured teeth, mobile teeth, and fractured teeth (Table 3).

**Table 2:** Frequency distribution of clinically examined oral health problems, by gender (N = 700)

Oral health problems (clinical)	Frequency N = 700	Gender		p value
		Boys 361 (51.6%)	Girls 339 (48.4%)	
*Decay prevalence (1* + *D)*				
Yes	241 (34.4%)	119 (33.0%)	122 (36.0%)	0.4
No	459 (65.6%)	242 (67.0%)	217 (64.0%)	
*Missing prevalence (1* + *M)*				< 0.001
Yes	142 (20.3%)	42 (11.6%)	100 (29.5%)	
No	558 (79.7%)	319 (88.4%)	239 (70.5%)	
*Filled prevalence (1* + *F)*				< 0.001
Yes	172 (24.6%)	34 (9.4%)	138 (40.7%)	
No	528 (75.4%)	327 (90.6%)	201 (59.3%)	
DMFT—mean (SD)	3.8 (0.75)	3.54 (0.63)	4.06 (0.77)	< 0.001
Decay severity (DT)—mean (SD)	1.31 (1.81)	1.29 (1.83)	1.33 (1.80)	0.7
Missing severity (MT)—mean (SD)	0.95 (1.38)	0.97 (1.25)	0.92 (1.50)	0.6
Filled severity (FT)—mean (SD)	1.46 (1.57)	1.22 (1.41)	1.73 (1.69)	< 0.001
*Gingival Index*				0.03
Normal gingiva	573 (81.9%)	303 (84%)	270 (79.6%)	
Mild inflammation	72 (10.3%)	39 (10.8%)	33 (9.7%)	
^1^Moderate/severe inflammation	55 (7.8%)	19 (5.2%)	36 (10.6%)	
*Plaque Index*				0.002
Absence of plaque	443 (63.3%)	251 (69.5%)	192 (56.6%)	
Mild accumulation	124 (17.7%)	52 (14.4%)	72 (21.2%)	
^1^Moderate/severe accumulation	133 (19%)	58 (16%)	75 (22.1%)	

^1^Responses of moderate/severe gingival inflammation and plaque deposits were merged due to lower proportion of boys and girls with severe condition

**Table 3:** Frequency distribution of self-perceived oral health problems, by gender (N = 700)

Oral health problems	Frequency N = 700	Gender		p value
		Boys 361 (51.6%)	Girls 339 (48.4%)	
Tooth decay	257 (36.7%)	111 (30.7)	146 (43.0%)	< 0.01
Tooth ache	231 (33.0%)	112 (31.0%)	119 (35.1%)	0.3
Tooth discoloration	172 (24.6%)	107 (29.6%)	65 (19.2%)	< 0.01
Tooth extraction	73 (10.4%)	10 (2.8%)	63 (18.6%)	0.00
Tooth sensitivity	70 (10.0%)	39 (10.8%)	31 (9.1%)	0.3
Tooth mobility	55 (7.9%)	44 (12.2%)	11 (3.2%)	0.00
Halitosis	46 (6.6%)	16 (4.4%)	30 (8.8%)	0.02
Abnormally shaped tooth	45 (6.4%)	21 (5.8%)	24 (7.1%)	0.5
Tooth extrusion	43 (6.1%)	21 (5.8%)	22 (6.5%)	0.7
Mal-aligned teeth	41 (5.9%)	18 (5%)	23 (6.8%)	0.3
Bleeding gums	41 (5.9%)	20 (5.5%)	21 (6.2%)	0.7
Swollen gums	39 (5.6%)	17 (4.7%)	22 (6.5%)	0.2
Fractured teeth	35 (5.0%)	27 (7.5%)	8 (2.6%)	< 0.01

About 40% of the children had at least one impacted daily performance (1 + performance) (Table 4). Only a few children had frequent, and none had a severely impacted daily performance. The mean oral impact score (OIS) was calculated for the overall sample and also for the impacted only. Both these values varied significantly, and the mean OIS was higher in the impacted only (Table 4); the OIS of 1 + performance of sample considering impacted only was twice the value (10.51) to that of the overall sample (5.25). Based on the prevalence score, study activity was the most reported impacted daily performance (38%) and also had higher mean oral impact scores (mean_overall sample_ = 1.4 and mean_impacted only_ = 3.9). The impact of OH conditions on speaking, cleaning teeth, emotion, and smiling performances were not reported in almost all the children,

**Table 4:** Frequency, prevalence, severity, and oral impact scores of the daily performances (N = 700)

Child-OIDP	Performances								
	1 + Performance	Eating	Speaking	Cleaning teeth	Sleeping	Emotion	Smiling	Study	Social contact
*Frequency in past 3 months* *n (%)*									
Not at all	420 (60.0)	545 (77.9)	699 (99.9)	699 (99.9)	486 (69.4)	699 (99.9)	699 (99.9)	429 (61.3)	553 (79.0)
Once	208 (29.7)	45 (6.4)	0	0	80 (11.4)	0	0	81 (11.6)	68 (9.7)
Two times	34 (4.9)	73 (10.4)	0	0	79 (11.3)	0	0	104 (14.9)	48 (6.9)
Three times or more	38 (5.4)	37 (5.3)	1 (0.1)	1 (0.1)	55 (7.9)	1 (0.1)	1 (0.1)	86 (12.3)	31 (4.4)
Prevalence n (%)	280 (40.0)	155 (22.1)	1 (0.1)	1 (0.1)	214 (30.6)	1 (0.1)	1 (0.1)	266 (38.0)	147 (21.0)
*Severity in past 3 months* *n (%)*									
Not at all	426 (60.9)	546 (78)	699 (99.9)	699 (99.9)	486 (69.4)	699 (99.9)	699 (99.9)	429 (61.3)	553 (79)
Little	218 (31.1)	24 (3.4)	0	0	67 (9.6)	0	0	54 (7.7)	112 (16)
Moderate	56 (8)	130 (18.6)	1 (0.1)	1 (0.1)	147 (21)	1 (0.1)	1 (0.1)	217 (31)	35 (5)
Severe	0	0	0	0	0	0	0	0	0
*Oral Impact Score*									
Range (minimum–maximum)	0–48	0–6	0–6	0–6	0–6	0–6	0–6	0–6	0–6
Mean (SD)	5.25 (8.36)	0.82 (1.73)	–	–	1.04 (1.89)	–	–	1.44 (2.17)	0.48 (1.19)
*Oral Impact Score (Impacted only)*									
Range (minimum–maximum)	0–24	0–6	0–6	0–6	0–6	0–6	0–6	0–6	0–6
Mean (SD)	10.51 (16.73)	3.73 (1.66)	–	–	3.40 (1.92)	–	–	3.88 (1.78)	2.31 (1.59)

The OIS for each daily performance was considered as the outcome variable and simple linear regressions were carried out to report the regression coefficients (RC). In general, dental caries, gingival and plaque indices from the clinical measures and; tooth decay and toothache from the self-perceived measures were significantly associated with the impacted daily performances (Table 5). The caries measures; DT, DMFT, 1 + D and self-perceived tooth decay were associated with greater OIS of 1 + performance, eating, sleeping, study, and social contact (RC_range_ = 0.3–13.7) and; the treatment measures MT, FT, and 1 + M were associated with lower OIS of daily performances (RC_range_ = − 1.9 to − 0.1). Poorer gingival and plaque statuses were also associated with greater OIS (RC_range_ = 0.5–7.7). Other self-perceived indicators associated with most of the impacted daily performances were toothache and tooth discolouration (Table 5).

**Table 5:** Simple linear regression for association between oral impact scores of daily performances and oral health measures (N = 700)

Independent variables	1 + Performance—OIS	Eating—OIS	Sleeping—OIS	Study—OIS	Social contact—OIS
	RC (95% CI)	RC (95% CI)	RC (95% CI)	RC (95% CI)	RC (95% CI)
*Clinical OHS*					
Decay severity (DT)	3.3 (3.09, 3.59)^b^	0.6 (0.51, 0.63)^b^	0.6 (0.59, 0.71)^b^	0.8 (0.75, 0.88)^b^	0.3 (0.31, 0.39)^b^
Missing severity (MT)	− 1.5 (− 1.97, − 1.07)^b^	− 0.3 (− 0.40, − 0.22)^b^	− 0.2 (− 0.35, − 0.15)^b^	− 0.4 (− 0.50, − 0.27)^b^	− 0.1 (− 0.20, − 0.07)^b^
Filled severity (FT)	− 1.9 (− 2.37, − 1.62)^b^	− 0.3 (− 0.42, − 0.26)^b^	− 0.3 (− 0.43, − 0.26)^b^	− 0.5 (− 0.59, − 0.40)^b^	− 0.3 (− 0.31, − 0.21)^b^
DMFT	5.4 (4.70, 6.19)^b^	0.7 (0.57, 0.89)^b^	1.4 (1.25, 1.56)^b^	1.3 (1.10, 1.48)^b^	0.4 (0.31, 0.53)^b^
Decay prevalence (1 + D)	13.3 (12.44, 14.24)^b^	2.2 (2.00, 2.43)^b^	2.7 (2.51, 2.94)^b^	3.2 (3.00, 3.48)^b^	1.3 (1.17, 1.48)^b^
Missing prevalence (1 + M)	0.6 (− 0.97, 2.19)	− 0.4 (− 0.69, − 0.05)^a^	0.6 (0.29, 0.98)^b^	0.04 (− 0.35, 0.44)	0.2 (− 0.05, 0.39)
Filled prevalence (1 + F)	− 0.3 (− 1.76, 1.19)	− 0.1 (− 0.45, 0.14)	0.4 (0.05, 0.70)^a^	− 0.1 (− 0.45, 0.29)	− 0.5 (− 0.69, − 0.29)^b^
Gingival index	5.9 (4.95, 6.91)^b^	0.6 (0.41, 0.83)^b^	1.1 (0.89, 1.33)^b^	1.9 (1.65, 2.15)^b^	0.5 (0.34, 0.63)^b^
Plaque index	7.7 (7.17, 8.30)^b^	1.4 (1.26, 1.51)^b^	1.6 (1.53, 1.79)^b^	1.8 (1.64, 1.94)^b^	0.6 (0.55, 0.75)^b^
*Self-perceived OHS*					
Tooth decay	13.7 (12.87, 14.55)^b^	2.2 (1.95, 2.38)^b^	2.8 (2.61, 3.02)^b^	3.6 (3.39, 3.79)^b^	1.2 (1.05, 1.37)^b^
Toothache	15.0 (14.26, 15.79)^b^	2.4 (2.25, 2.65)^b^	3.1 (2.96, 3.34)^b^	3.8 (3.58, 3.97)^b^	1.3 (1.17, 1.49)^b^
Tooth extraction	− 1.4 (− 3.52, 0.64)	− 0.7 (− 1.16, − 0.33)^b^	0.03 (− 0.43, 0.49)	− 0.10 (− 0.62, 0.43)	− 0.2 (− 0.48, 0.10)
Tooth sensitivity	− 0.05 (− 2.17, 2.07)	− 0.3 (− 0.73, 0.12)	− 0.2 (− 0.69, 0.25)	0.3 (− 0.20, 0.87)	0.2 (− 0.10, 0.49)
Tooth discoloration	2.3 (0.81, 3.74)^a^	0.7 (0.40, 0.98)^b^	0.5 (0.14, 0.79)^a^	0.2 (− 0.19, 0.56)	0.3 (0.14, 0.55)^a^
Fractured teeth	− 3.1 (− 6.07, − 0.25)^a^	− 0.5 (− 1.08, 0.09)	− 0.7 (− 1.32, 0.03)^a^	− 0.7 (− 1.38, 0.09)	− 0.4 (− 0.82, − 0.02)^a^
Abnormal shaped	− 1.05 (− 3.64, 1.54)	− 0.2 (− 0.78, 0.27)	− 0.3 (− 0.92, 0.22)	− 0.1 (− 0.80, 0.51)	0.03 (− 0.33, 0.39)
Mal-aligned teeth	− 0.4 (− 3.16, 2.26)	− 0.1 (− 0.69, 0.40)	0.3 (− 0.33, 0.87)	− 0.4 (− 1.08, 0.29)	− 0.02 (− 0.40, 0.35)
Bleeding gums	− 1.8 (− 4.52, 0.89)	− 0.5 (− 1.05, 0.01)	− 0.5 (− 1.11, 0.09)	− 0.2 (− 0.84, 0.53)	− 0.1 (− 0.48, 0.28)
Swollen gums	− 1.5 (− 4.31, 1.23)	− 0.4 (− 0.99, 0.13)	− 0.5 (− 1.09, 0.13)	− 0.09 (− 0.79, 0.61)	− 0.1 (− 0.46, 0.31)
Halitosis	− 0.02 (− 2.59, 2.55)	− 0.2 (− 0.74, 0.29)	− 0.1 (− 0.66, 0.48)	0.4 (− 0.21, 1.08)	− 0.1 (− 0.46, 0.26)
Tooth mobility	− 3.0 (− 5.37, − 0.66)^a^	− 0.8 (− 1.28, − 0.33)^a^	− 0.3 (− 0.82, 0.22)	− 0.7 |(− 1.27, − 0.08)^a^	− 0.03 (− 0.67, 0.02)^a^
Tooth extrusion	0.5 (− 2.14, 3.16)	− 0.2 (− 0.71, 0.36)	0.1 (− 0.50, 0.67)	− 0.05 (0.72, 0.62)	− 0.04 (− 0.41, 0.32)
*SD*					
Gender (Male)	2.0 (0.78, 3.31)^a^	0.2 (− 0.01, 0.50)	0.4 (0.08, 0.64)^a^	0.6 (0.25, 0.88)^a^	0.2 (0.05, 0.40)^a^
Location (Rural)	− 2.6 (− 3.86, − 1.31)^b^	− 0.4 (− 0.63, − 0.12)^a^	− 0.4 (− 0.65, − 0.09)^a^	− 0.8 (− 0.11, − 0.47)^b^	− 0.4 (− 0.56, − 0.21)^b^
Mothers education	− 0.8 (− 1.58, − 0.01)^a^	− 0.02 (− 0.18, 0.14)	− 0.2 (− 0.42, − 0.07)^a^	− 0.2 (− 0.41, − 0.02)^a^	− 0.1 (− 0.26, − 0.04)^a^
Fathers education	1.5 (0.46, 2.49)^a^	0.5 (0.30, 0.71)^b^	0.1 (− 0.12, 0.33)	0.2 (− 0.03, 0.48)	0.1 (− 0.04, 0.24)
*OHB*					
Mode of brushing					
Miswak	-.3.7 (− 5.54, − 1.93)^b^	− 0.6 (− 0.99, − 0.27)^a^	− 0.7 (− 1.12, − 0.32)^b^	− 0.8 (− 1.34, − 0.42)^b^	− 0.4 (− 0.67, − 0.17)^a^
Toothbrush	4.4 (2.82, 5.99)^b^	0.7 (0.44, 1.08)^b^	0.9 (0.55, 1.25)^b^	0.9 (0.58, 1.38)^b^	0.5 (0.27, 0.71)^b^
Both	− 4.8 (− 7.68, − 1.89)^a^	− 0.8 (− 1.44, − 0.27)^a^	− 1.1 (− 1.74, − 0.45)^a^	− 0.9 (− 1.68, − 0.21)^a^	− 0.5 (− 0.91, − 0.11)^a^
Frequency of brushing	− 6.5 (− 7.35, − 5.75)^b^	− 0.9 (− 1.10, − 0.75)^b^	− 1.3 (− 1.49, − 1.12)^b^	− 1.9 (− 2.08, − 1.70)^b^	− 0.6 (− 0.71, − 0.47)^b^
Dental visit	3.0 (2.48, 3.52)^b^	0.5 (0.41, 0.63)^b^	0.7 (0.54, 0.78)^b^	0.7 (0.53, 0.80)^b^	0.2 (0.18, 0.33)^b^

^a^p value < 0.05

^b^p value < 0.001

Association findings of demographic characteristics showed that an increase in the education level of mothers and children living in rural areas, were associated with lower OIS. For oral hygiene behaviours; children using miswak exclusively and, using both miswak and toothbrush had lower OIS (RC_range_ = − 4.8 to − 0.4). Similarly, an increase in the frequency of brushing was associated with lower OIS (RC_range_ = − 6.5 to − 0.6). Lastly, children with less frequent visit to the dentists were associated with greater OIS (RC_range_ = 0.5 to 3.0).

The results of multiple linear regression analyses are presented in Table 6. Based on the magnitude and precision of adjusted regression coefficients (RC), the clinical OH measure that was identified as a better predictor of greater OIS was decay severity (DT), with RC values ranging between 0.3 (social contact) and 2.4 (1 + performance). The DMFT (Adjusted RC_range_ = 0.2 to 3.7) was the next best predictor, however, DMFT includes MT and FT components which were associated with lower OIS. Overall, the self-perceived measures were also associated with OIS and presented similar findings to that of the clinical measures. The tooth decay, toothache, and tooth discolouration were associated with greater OIS in all or most of the daily performances; whereas, tooth extraction, tooth sensitivity, fractured teeth, and tooth mobility were associated with lower OIS (Table 6).

**Table 6:** Multiple linear regression for the effects of oral health measures on the oral impact scores of daily performances after adjusting for the significant covariates (N = 700)

Decay severity	Adjusted RC (95% CI)				
	1 + Performance—OIS	Eating- OIS	Sleeping- OIS	Study- OIS	Social contact- OIS
Decay severity (DT)	2.4 (2.15, 2.68)^2^	0.4 (0.39, 0.52)^2^	0.4 (0.39, 0.52)^2^	0.5 (0.46, 0.59)^2^	0.3 (0.23, 0.32)^2^
Gender (male)	1.3 (0.31, 1.94)^1^	–	–	0.3 (0.11, 0.51)^1^	0.1 (0.00, 0.30)^1^
Location (rural)	− 2.1 (− 2.95, − 1.22)^2^	− 0.2 (− 0.44, − 0.02)^1^	− 0.3 (− 0.53, − 0.10)^1^	− 0.8 (− 0.99, − 0.57)^2^	− 0.3 (− 0.44, − 0.13)^2^
Frequency of brushing	− 3.7 (− 4.41, − 3.07)^2^	− 0.4 (− 0.55, − 0.22)^2^	− 0.7 (− .0.91, − 0.57)^2^	− 1.3 (− 1.49, − 1.16)^2^	− 0.3 (− 0.42, − 0.18)^2^
Dental visit	1.2 (0.94, 1.61)^2^	0.2 (0.13, 0.32)^2^	0.3 (0.23, 0.43)^2^	0.2 (0.13, 0.32)^2^	–

OH factors	Adjusted RC (95% CI)^1^				
	1 + Performance—OIS	Eating—OIS	Sleeping—OIS	Study—OIS	Social contact—OIS
*Clinical problems*					
Decay severity (DT)	2.4 (2.15, 2.68)^2,GLFD^	0.4 (0.37, 0.50)^2,EFD^	0.5 (0.39, 0.52)^2,EFDLZG^	0.5 (0.46, 0.59)^2,DFZMLG^	0.3 (0.22, 0.32)^2,DFZMXLG^
Missing severity (MT)	− 0.9 (− 1.28, − 0.58)^2,LFD^	− 0.2 (− 0.28, − 0.10)^2,EFDL^	− 0.1 (− 0.20, − 0.03)^1,FDLZ^	− 0.2 (− 0.31, − 0.14)^2,DFZXLG^	− 0.10 (− 0.15, − 0.03)^2,DFZMXL^
Filled severity (FT)	− 1.1 (− 1.38, − 0.73)^2,GLFD^	− 0.2 (− 0.26, − 0.12)^2,EFDLZ^	− 0.1 (− 0.23, − 0.10)^2,FDLZ^	− 0.2 (− 0.31, − 0.16)^2,DFZLG^	− 0.2 (− 0.24, − 0.13)^2,DFZXLG^
Decay prevalence (1 + D)	10.0 (8.99, 10.96)^2,GLFD^	1.7 (1.42, 1.94)^2,EFDL^	2.0 (1.79, 2.30)^2,FDLZ^	2.2 (1.96, 2.43)^2,DFZMLG^	1.0 (0.83, 1.20)^2,DFZXLG^
Missing prevalence (1 + M)	–	− 0.3 (− 0.57, − 0.11)^1,EFDL^	0.6 (0.28, 0.85)^2,FDLZ^	–	–
DMFT	3.7 (3.03, 4.39)^2,GLFD^	0.5 (0.32, 0.65)^2,EFDLM^	1.2 (1.10, 1.38)^2,FDLZMG^	0.8 (0.66, 0.98)^2,DFZML^	0.2 (0.10, 0.32)^2,DFZXL^
Gingival index	2.0 (1.21, 2.76)^2,GLFD^	0.5 (0.33, 0.75)^2,EMT^	0.4 (0.23, 0.64)^2,FDLZ^	1.1 (0.88, 1.26)^2,DFZXLG^	0.2 (0.05, 0.34)^1,DFZXL^
Plaque index	5.3 (4.76, 5.87)^2,LFD^	1.2 (1.08, 1.34)^2,EMFL^	1.3 (1.21, 1.49)^2,FDLZ^	1.3 (1.15, 1.43)^2,DFZL^	0.5 (0.41, 0.61)^2,FZXL^
–					
Tooth decay	11.1 (10.13, 12.16)^2,LFD^	1.8 (1.59, 2.10)^2,EMFL^	2.4 (2.16, 2.62)^2,FDZ^	2.7 (2.45, 2.91)^2,DFZML^	0.8 (0.65, 1.05)^2,DFZXL^
Toothache	12.8 (11.87, 13.70)^2,GLFD^	2.3 (2.06, 2.48)^2,EML^	2.8 (2.60, 3.03)^2,FDZG^	2.9 (2.65, 3.10)^2,DFZMLG^	1.0 (0.84, 1.24)^2,DFXLG^
Tooth extraction	–	− 0.4 (− 0.77, − 0.01)^1,EFDL^	–	–	–
Tooth sensitivity	–	− 0.5 (− 0.84, − 0.11)^1,EFDL^	− 0.4 (− 0.80, − 0.02)^1,FDLZ^	–	–
Tooth discoloration	2.0 (0.78, 3.19)^1,LFD^	0.3 (− 0.59, − 0.11)^1,EFD^	0.5 (0.18, 0.75)^1,FDLZM^	–	0.3 (0.13, 0.53)^1,DFZMXLG^
Fractured teeth	− 2.9 (− 5.76, 0.01)^1,L^	–	− 0.7 (− 1.32, 0.03)^1^	^−^	− 0.4 (− 0.82, − 0.02)^1^
Tooth mobility	− 3.5 (− 5.84, − 1.16)^1,L^	− 0.5 (− 0.96, − 0.10)^1,EFL^	–	− 0.9 (− 1.53, − 0.36)^2,MXLG^	− 0.3 (− 0.62, − 0.01)^1,FMXL^

^1^p value < 0.05

^2^p value < 0.001

^F^Frequency of brushing; ^D^Dental visits; ^T^Toothbrush; ^M^Miswak; ^Z^Miswak and Toothbrush; ^X^Mothers education; ^E^Fathers education; ^L^Location; ^G^Gender

The multiple regression analysis was not performed because the independent variable was not significant in the binary regression analysis

## Discussion

The present study identified the oral health (OH) conditions and their measures that are associated with greater oral impact scores (OIS) of the daily performances, and the investigation included a wide range of OH conditions that commonly affect adolescent-aged children [21]. This approach of using OIS rather than presence or absence of impact as the outcome variable is unique. Despite OIS being stated as a comprehensive measure by the founders of C-OIDP and includes prevalence, frequency, and severity of impacts [4], no earlier report had used it as the patient reported outcome measure. There is a difference in analyses and interpretation; for instance, Kumar and colleagues carried out logistic regression to state that children with caries had about 6-times greater chance of having an impact on daily performance than caries-free children [22]. Contrastingly, in this study through linear regression, it is shown that by a unit increase in decay severity (DT) the oral impact score (OIS) will increase by two units.

About 40% of the children in the current study reported at least one impacted daily performance. Taking this into account, the current study refined the method of calculating OIS by Gherunpong et al. [4] and states that differentiation should be drawn between the report from all samples and those who reported an impact. Findings from this study demonstrate that the mean value is refined if only the children who had reported to be impacted are considered instead of the whole study sample. For instance, using the original method by Gherunpong and colleagues [4], the impact on the study had the highest mean OIS among the eight performances (1.44 ± 2.17). However, by considering only the children who reported an impact the mean OIS for study performance increased noticeably (3.88 ± 1.78).

Impacted study or school-work was reported by a majority (38%) of the children in this study, and it was associated with several self-perceived and clinically assessed OH measures. This finding is consistent with studies performed in the United States [23, 24], Thailand [4, 25], Malaysia [8] and Indonesia [26]. However, the methodology of some studies [23, 26] has been questioned and discussed in a published commentary [27]. Further evidence from explicit investigations could offer a comprehensive understanding on the relationship between OH conditions and school performance.

Caries related clinical and self-perceived measures are observed to be consistent in demonstrating an association with oral impact scores for each daily performance and the 1 + performance. However, measures related to treatment, such as missing and filled teeth prevalence (1 + M and 1 + F) and severity (MT and FT), and self-perceived tooth extraction showed protective effects. From caries measures related to untreated disease condition, such as 1 + D, DT, and toothdecay, the clinical OH measures examined and reported by trained dentists are fairly reliable. This is because, OH conditions diagnosed by children may depend on factors such as oral health literacy, whereby, children with better OH literacy are more likely to provide an accurate response [28]. Between the two clinical measures related to untreated caries i.e. DT and 1 + D, the former provided better estimation of the association with reduced standard errors and narrower confidence intervals. This finding was consistent in all the analysed models having oral impact scores of each impacted daily performances as the outcome variables. These appraisals of OH measures are exclusive, and therefore, the findings cannot be explicitly compared to the earlier reports. Moreover, the earlier investigations involving clinical examinations were carried out on a comparitively younger population (less than 9 years of age) and the responses of questions pertaining to OHRQoL were obtained from parents or guardians [29, 30], and were performed on children with special needs [30, 31]. Karki et al. reported their findings using the same C-OIDP questionnaire for 5–6-year-olds, 12-year-olds and 15-year-olds [9], when the original version was constructed and validated for children of age 11 years and older [4], suggesting that the C-OIDP protocol was not followed.

As secondary findings, the current study reveals that nearly 34% of the children had untreated caries, and this finding was consistent with previous studies performed in the same region [32–34]. The risk of sepsis and pain from a decayed tooth is considerably greater, and Marcenes et al. concluded that if decay among children is not prevented then the burden of disease will further affect the life events of children as well as impact heavily on the economy of a nation via the cost of treatment services [35].

There are several strengths of the current study. The findings were derived from a homogenized population and analyses controlled for demographics such as age, gender, and parental education. The assessment of impacted daily performances was performed using language validated [6] and culturally tested [36] C-OIDP questionnaire. Also, a wide variety of the OH conditions were considered using the clinical observations and self-perception to determine the OH conditions and their measures that influence daily performances of schoolchildren [37]. However, the current study does not come without limitations. As the design is cross-sectional, the causal association between the OH conditions and the impacted daily performances cannot be established. Impacts on speaking, cleaning teeth, emotion, and smiling performances were not reported in almost all the children, and this common response from children is supported by the evidence that *respondents in a cluster tend to have similar views on OH and behaviour* and is similar to another published report [38].

In conclusion, the untreated tooth decay significantly impacted the daily performances of school-going children, especially their eating, sleeping, study, and social contact. Decay severity demonstrated more precise results in comparison to other oral health measures. Besides, the disease experience measures and the treatment measures showed opposing results. These findings are of importance to researchers, policymakers, oral health providers, and public health analysts; for understanding, reporting, treating, and preventing persistent OH conditions among the school-going children, and eventually contributing towards better oral health-related quality of life.

## Data Availability

The data for the current study will be made available upon reasonable request.
